# The m6A-induced lncRNA CASC8 promotes proliferation and chemoresistance via upregulation of hnRNPL in esophageal squamous cell carcinoma

**DOI:** 10.7150/ijbs.71234

**Published:** 2022-07-18

**Authors:** Qingnan Wu, Hongyue Zhang, Di Yang, Qingjie Min, Yan Wang, Weimin Zhang, Qimin Zhan

**Affiliations:** 1 Key Laboratory of Carcinogenesis and Translational Research (Ministry of Education/Beijing), Laboratory of Molecular Oncology, Peking University Cancer Hospital & Institute, Beijing 100142, China; 2 Shenzhen Bay Laboratory, Shenzhen 518132, China; 3 Research Unit of Molecular Cancer Research, Chinese Academy of Medical Sciences, Beijing 100021, China

**Keywords:** lncRNA, esophageal squamous cell carcinoma, cell apoptosis, m6A demethylation, CASC8

## Abstract

Long noncoding RNAs (lncRNAs) are dysregulated in many cancers. Here, we identified the molecular mechanisms of lncRNA Cancer Susceptibility Candidate 8 (CASC8) in promoting the malignancy of esophageal squamous cell carcinoma (ESCC). CASC8 was highly overexpressed in ESCC tissues and upregulation of CASC8 predicted poor prognosis in ESCC patients. Moreover, CASC8 decreased the cisplatin sensitivity of ESCC cells and promoted ESCC tumor growth *in vivo*. Mechanistically, CASC8 interacted with heterogeneous nuclear ribonucleoprotein L (hnRNPL) and inhibited its polyubiquitination and proteasomal degradation, thus stabilizing hnRNPL protein levels and activating the Bcl2/caspase3 pathway. Additionally, AlkB Homolog 5, RNA demethylase (ALKBH5)-mediated m6A demethylation stabilized the CASC8 transcript, resulting in CASC8 upregulation. Taken together, these findings identified an oncogenic function of CASC8 in the progression of ESCC, which suggest that CASC8 might become a potential prognostic biomarker in ESCC.

## Introduction

Esophageal carcinoma (EC) is one of the most frequent digestive cancers worldwide 1. The majority subtype of EC is esophageal squamous cell carcinoma (ESCC), which occurs widely in China 2, 3. The low five-year survival rate and the high mortality rate of ESCC are still prevalent, because of lacking effective methods for early diagnosis and therapy 4, 5. Therefore, the clinical curative efficacy of ESCC treatment should be improved urgently. It has been found that plenty of risk factors are closely related with the formation and progression of ESCC, such as activated oncogene, inactivated tumor suppressor. Especially, the epigenetic abnormalities such as acetylation and methylation are reported to play essential roles in the malignant progress of ESCC 6, 7, however the underlying molecular mechanisms of ESCC tumorigenesis are still remained unknown.

In the last decade, the increasing transcriptome studies have displayed that most human genes encode for noncoding RNA (ncRNA), other than proteins 8, 9. The ncRNA genes, particularly long ncRNA (lncRNA) genes, have been proven to link to various pathological development of cancers including ESCC. For example, lncRNA Linc01503 10, POU3F3 11, CCAT1 12 and so on were highly upregulated in ESCC and act an promoting role in tumor progression. The function studies of these lncRNAs attracted new insight for the therapeutic strategies of ESCC.

Using whole-genome sequencing technology, our group has comprehensively reported genomic alterations and identified that the region 8q24.21 was significantly amplified in ESCC 13, 14. The Cancer Susceptibility Candidate 8 (CASC8), which located at 8q24.21, showed significantly copy number amplification. The single-nucleotide polymorphisms of CASC8 have been found to be associated with the risk of EC 15, hepatocellular carcinoma 16 and colorectal cancer 17. Moreover, CASC8 exhibited oncogenic role in promoting cell proliferation in bladder cancer 18 and retinoblastoma 19. However, the molecular mechanism of CASC8 in tumorigenesis needs to be explored.

Here, we figured out an oncogenic role for lncRNA CASC8, which is frequently upregulated in ESCC tissues. Mechanistically, we demonstrated that the decrease in *N*6-methyladenosine (m^6^A)-modified RNA catalyzed by the m6A demethylase AlkB Homolog 5, RNA Demethylase (ALKBH5) induced the upregulation of CASC8, which subsequently promoted ESCC progression through the post-translational upregulation of hnRNPL.

## Materials and Methods

### Reagents

The primary antibodies of anti-hnRNPL (ab6106), IgG (ab171870) were from Abcam (Cambridge, MA, USA); the primary antibodies of anti-ALKBH5 (16837-1-AP), anti-β-actin (66009-1-g) and anti-METTL3 (15073-1-AP) were from Proteintech (Wuhan, China); the primary antibodies of anti-Bcl2 (3498S), anti-caspase3 (9665S), anti-caspase9 (9502S) and anti-Ubiquitin (3933S) were from Cell Signaling Technology (Danvers, MA, USA). Cisplatin, Actinomycin D, Cycloheximide and MG132 were purchased from MedChemExpress (Monmouth Junction, NJ, USA). Antisense LNA^TM^ GapmeRs for CASC8 were synthesized by QIAGEN (Frankfurt, Germany). siRNAs for CASC8, hnRNPL, and ALKBH5 were synthesized by Ribobio (Guangzhou, China).

### Cell lines and transfection

The human ESCC cell lines: COLO-680N, KYSE-510, 450, 410, 180, 150, 30 and YES-2 were obtained from Dr. Y. Shimada of Kyoto University. The cells were cultured in RPMI 1640 (Lonza, Basel, Switzerland) with 10% fetal bovine serum (Gibco, Invitrogen, Carlsbad, USA) at 37 °C in a 5% CO_2_ humidified incubator. The Antisense LNA^TM^ GapmeRs were used to silence the expression of CASC8. The small interfering RNAs (siRNAs) are used to inhibit the expressions of hnRNPL and ALKKBH5. Lipofectamine 2000 transfection reagent (Invitrogen) was used to transfect plasmid and siRNA/GapmeR following the instruction. The detailed target sequences are showed below:

GapmeR/CASC8-1 sequence: CGGTAACCTAGATTGG;

GapmeR/CASC8-2 sequence: TCGTTGGTAGGCTAGT.

si/hnRNPL-1 target sequence: GACGGGTCTTGCAGTTACA;

si/hnRNPL-2 target sequence: GTCAGTCATACGGGTTGGA.

si/ALKBH5-1 target sequence: GATCGCCTGTCAGGAAACA;

si/ALKBH5-2 target sequence: GTCCTTCTTTAGCGACTCT.

Lentivirus-mediated transfection with pLenti-U6-CASC8-shRNA-GFP-Puro and its empty vector were used for stably knockdown of CASC8. Lentivirus-mediated transfection with pCDH-CMV-CASC8-EF1-CopGFP-P2A-Puro and its empty vector were used for stably overexpression of CASC8.

### Tissue specimens

The tissue microarrays of ESCC were provided by Shanghai Outdo Biotech (HEsoS180Su09, Shanghai, China). All the patients who provides the tissues has signed the written informed consent. The study of these clinical specimens was permitted by Peking University Cancer Hospital Institutional Research Ethics Committee.

### *In situ* hybridization

The DNA probes complementary to CASC8 were labeled with digoxigenin and synthesized by random primer labeling (Boster, Wuhan, China). The proportion of positive staining tumor cells was graded: < 5%, 0; 5-25%, 1; 26-50%, 2; 51-75%, 3 and > 75%, 4. The intensity of positive staining tumor cells was graded as follows: 0, negative; 1, weak; 2, moderate; and 3, strong. Final scores were the result of the multiplication of these two primary scores. Final scores of 0-6 were defined as low expression and scores of 6-12 were defined as high expression 20.

### RNA isolation and qRT-PCR analyses

TRIzol reagent (Invitrogen) was used to isolated the total RNA from the cells and tissues following the manufacturer's protocol. The real-time quantitative polymerase chain reaction (qRT-RCR) assay was used to evaluate the gene expression. The reverse transcription kit (Takara, Dalian, China) was used to reverse the isolated RNA into cDNA and conducted in an ABI 7500 apparatus (Applied Biosystems, Foster City, CA, USA). The relative expression levels were normalized to the GAPDH expression. The PCR primers were as follows:

CASC8 F, 5′-CTGCTGATCATGTGCATAAGCC-3′; R, 5′-TGCTACATAACAGCCACCCAA-3′;

hnRNPL F, 5′-GTGTGGTGGAAGCAGACCTTGT-3′; R, 5′-CAAACTCCACCAGTGCTTGTCTC-3′;

18s F, 5′-CAGCCACCCGAGATTGAGCA-3′; R, 5′-TAGTAGCGACGGGCGGTGTG-3′;

U6 F, 5′-CTCGCTTCGGCAGCACA-3′; R, 5′-AACGCTTCACGAATTTGCGT-3′;

GAPDH F, 5′-CAATGACCCCTTCATTGACC-3′; R, 5′-TGGAAGATGGTGATGGGATT-3′.

### Isolation of cytoplasmic and nuclear RNAs

The PARIS^™^ Kit (AM1921, Thermo Fisher Scientific, Scotts Valley, USA) was used to isolated cytoplasmic and nuclear RNAs according to the manufacturer's instructions. The cytoplasmic and nuclear RNAs were analyzed by qRT-RCR assay.

### RNA fluorescence *in situ* hybridization (FISH)

The CASC8 probes were conjugated with fluorescence (Ribobio, Guangzhou, China) and applied for RNA-FISH as previously described 21. The cells were seeded on the thick glass and fixed with ice-cold 4% paraformaldehyde. Hybridization was performed using DNA probe sets according to the manufacturer's instructions. Images were captured and visualized by confocal microscope (ST2, Leica, Wetzlar, Germany).

### Cell viability assay

The cell viability was measured by Cell Titer 96^®^ AQueous One Solution Cell Proliferation Assay (Promega, Madison, WI, USA). Briefly, 5 × 10^3^ treated cells were seeded in the 96-well plate, 10% MTS in PBS was added and incubated at 37 °C for 1 h. The MTS solution absorbance was collected at 490 nm by infinite M200pro (TECAN, Hombrechtikon, Swiss).

### Bromodeoxyuridine (BrdU) incorporation

5 × 10^3^ transfected cells per well were plated into 96-well plates and incubated with BrdU for 24 h. Then the cells were fixed using ice-cold 70% ethanol for 5 min and washed with PBS. The immunostaining steps was performed according to the manufacturer's protocol. Finally, the plate was read at dual wavelengths of 450/550 nm by infinite M200pro (TECAN).

### Western blot analysis

RIPA buffer (Beyotime, Shanghai, China) with protease inhibitors and phosphatase inhibitor was used to extracted the total protein. The sample was incubated on ice and vortexed for 5 min. The lysates were centrifuged at 12,000 × *g* for 15 min and collected the supernatant. Protein concentrations were determined by the Pierce BCA protein assay kit (Thermo). 20 μg of total protein were subjected to an SDS-polyacrylamide gel and transferred to PVDF membranes. The membranes were blocked in 5% non-fat dry milk blocking buffer for 1 h and incubated with antibodies at 4°C overnight. The Amersham Imager 600 (Amersham, Chalfont, UK) was used to detect chemiluminescence signals.

### Colony formation assay

2 × 10^3^ of transfected cells were placed in 6-well plates. After 10 days, the cell colonies were washed with PBS and fixed with methyl alcohol for 5 min. Then the 6-well plates were stained with Crystal Violet.

### The tumor formation assay in nude mice

The male BALB/c nude mice (Vital River Laboratories, Beijing, China) were subcutaneously injected 1 × 10^6^ cells/mL of transfected ESCC cells at the right flanks. The tumor volumes were evaluated by the equation, V = 1/2× length × width^2^. Tumor growth was measured weekly, and mice were euthanized one month after injection. The tumor growth was determined by Ki67 staining and Hematoxylin and eosin staining. The animal experiment was approved by the experimental animal department of Peking university cancer hospital.

### Protein stability and mRNA stability assay

Cells were treated with 100 μg/ml cycloheximide (CHX) for indicated time to study protein stability by western blot. Cells were treated with 5 μg/ml actinomycin D (actD) for indicated time to study mRNA stability by qRT-PCR.

### RNA pull-down assay

The Magnetic RNA-Protein Pull-Down kit was used for RNA pull-down assay following the manufacturer's instructions (Pierce, Waltham, MA, USA). The RiboMAX^™^ Large Scale RNA Production System was used to synthesize the full-length CASC8 (Promega, Madison, WI, USA). Then the biotin-labelled CASC8 was pre-incubated with the beads and the cell protein lysate was added for incubating overnight. The beads were washed three times and denaturalized in loading buffer. The CACS8 binding protein was analyzed by western blot.

### RNA immunoprecipitation (RIP) assay

The Magna RIP RNA-Binding Protein Immunoprecipitation kit (Merck Millipore, Oakville, Ontario, Canada) was used for RIP assay following the manufacturer's instructions 22. The hnRNPL antibodies were used to pull down the target RNA and and IgG was used as negative control. The qRT-PCR assay was performed to show the fold enrichment of CASC8.

### Measurement of Total m^6^A in cells

Total m^6^A content was measured in 200 ng aliquots of total RNA extracted from Het-1A and KYSE-450 cells using an m^6^A RNA methylation quantification kit (Epigentek) according to the manufacturer's instructions.

### Methylated RNA immune‑precipitation (MeRIP) assay

Total RNA was isolated from KYSE-450 cells. According to the instruction of Magna methylated RNA immune-precipitation (MeRIP) m6A Kit (Merck Millipore), the fragmented RNAs were immunoprecipitated by the m^6^A antibody. qRT-PCR assay was used to analyze enrichment of m^6^A containing RNA.

### Statistical analysis

The correlation of CASC8 protein levels between cancer and adjacent normal tissue, or various clinical parameters was analyzed by chi-square test. Kaplan-Meier method and compared log-rank test were used to analyze overall survival. Statistical analysis was performed with one-way ANOVA or the 2-tailed Student's *t*-test. All data were obtained from at three independent experiments and showed as the mean ± SEM. All the statistical analysis was performed by SPSS software (version 25.0). A value of P < 0.05 was considered statistically significant.

## Results

### The upregulated CASC8 in ESCC predicts a poor prognosis

**Figure 1:**
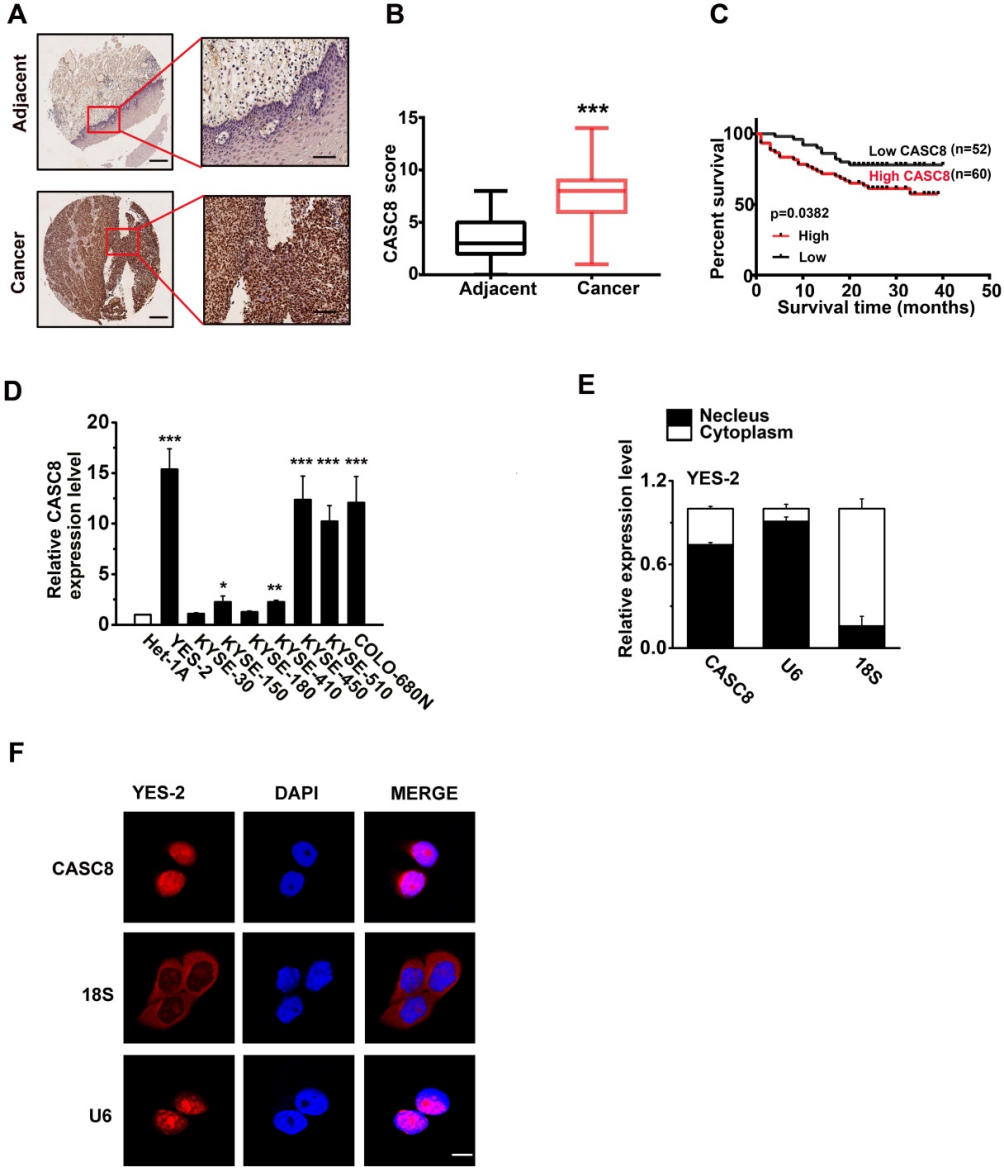
The expression of CASC8 in esophageal squamous cell carcinoma. **(A)** The *in suit* hybridization showed the CASC8 RNA expression in tissues. Scale bar, left, 100μm; Right, 200μm. **(B)** The CASC8 staining scores in ESCC tissue and adjacent normal tissues. **(C)** The survival time of patients with high levels of CASC8 expression was shorter than patients with low levels of CASC8 expression (P=0.0382, log-rank test). **(D)** Expression of CASC8 in eight ESCC cell lines and in the normal esophageal epithelial cell Het-1A cell. **(E)** Representative analysis of the CASC8 cellular distribution in YES-2 cells by qRT-PCR. U6 mRNA was control for nuclear RNAs and 18s mRNA was control for cytoplasmic RNAs. **(F)** The expression of CASC8 in YES-2 cells detected by RNA-FISH. DAPI staining represented the nuclei. Scale bar, 10μm. *P<0.05, **P<0.01, and ***P<0.001. All of the values are expressed as the mean ± SEM.

We previously analyzed the copy number alterations in ESCC and identified that the region 8q24.21 was significantly amplified 13, 14. Intriguingly, we found that the lncRNA CASC8, which located in 8q24.21, had remarkable high frequency amplification (40%) in ESCC after analysis of our previously published data. Then, we analyzed the CASC8 in public database CCLE (https://sites.broadinstitute.org/ccle) and confirmed that CASC8 was highly expressed in esophageal carcinoma cell lines (Figure S1). For further validation, we examined the CASC8 expression in ESCC tissues by *in situ* hybridization assay. The ESCC tissue microarrays contained 112 cases of ESCC and 68 cases of normal esophageal tissue. CASC8 staining was dark brown in the ESCC whereas it was nearly undetectable in most normal tissues (Figure 1A). These results indicated that CASC8 was significantly upregulated in ESCC tissues, comparing to the adjacent normal tissues (Figure 1B). Moreover, ESCC patients with high expression level of CASC8 were related with poorer prognosis (P=0.0382; Figure 1C). Next, we explored the relationship between CASC8 expression levels and a various of clinicopathological parameters of ESCC patients. CASC8 expression was positively correlated with pathological grade (Table 1). In addition, the eight ESCC cell lines displayed high CASC8 expression, while the normal esophageal epithelial cell line Het-1A displayed the lowest CASC8 expression (Figure 1D). We then analyzed the subcellular localization of CASC8 in YES-2 cells by performed cellular fractionation assay. CASC8 was found mostly in the nucleus of YES-2 cells (Figure 1E). Further confirmation through RNA-FISH assay displayed that CASC8 expression was largely located in the nucleus in ESCC cells (Figure 1F).

### CASC8 regulates cell proliferation and cisplatin sensitive in ESCC

**Figure 2:**
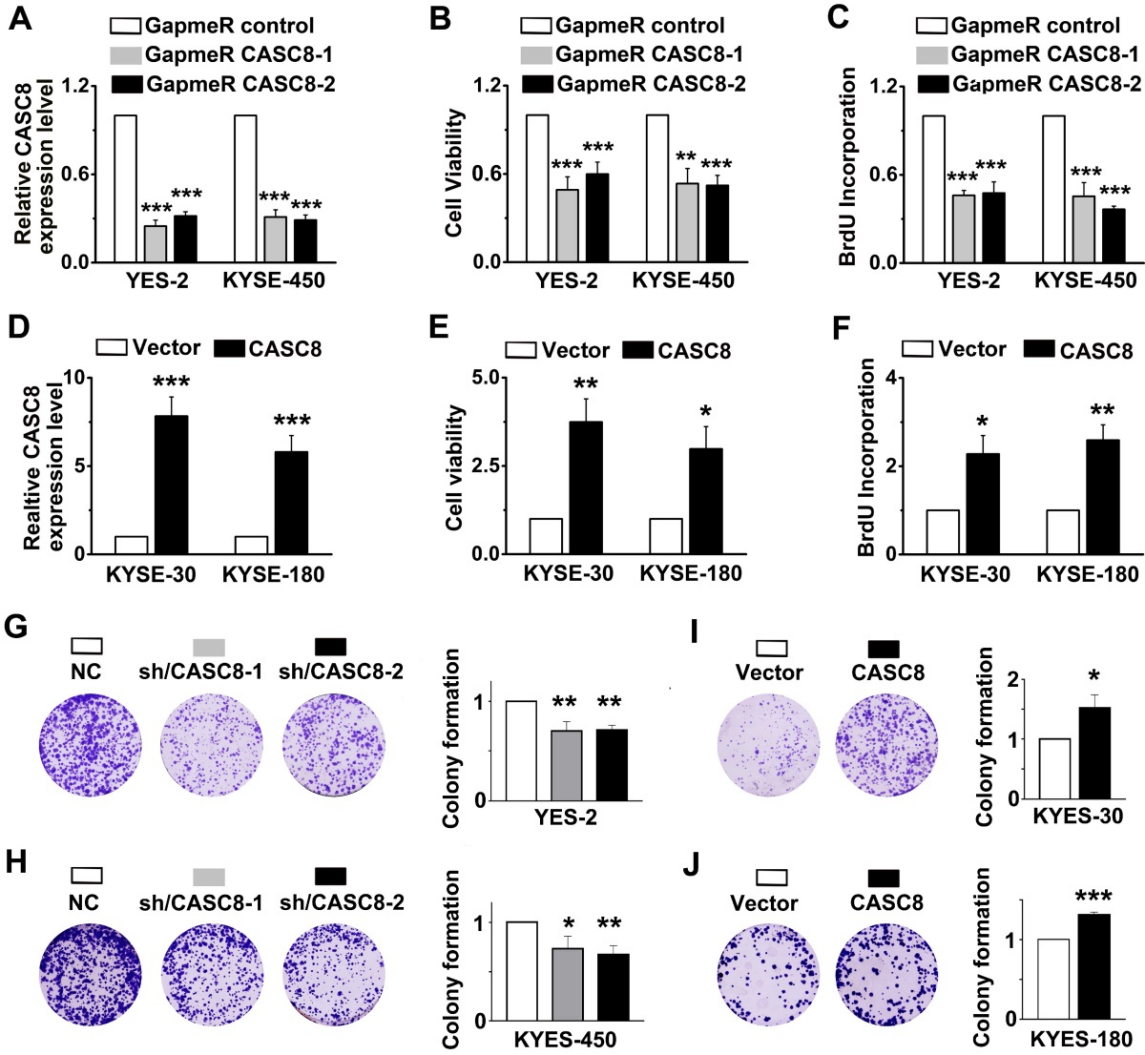
CASC8 regulates cell proliferation in ESCC. **(A)** CASC8 silencing in YES-2 and KYSE-450 cells after transfection with GapmeR CASC8. **(B)** The cell viability was determined by MTS assay in YES-2 and KYSE-450 cells. **(C)** The synthesis of DNA was showed by BrdU incorporation assay in YES-2 and KYSE-450 cells. **(D)** The overexpression efficiency of CASC8 in KYSE-180 and KYSE-30 cells. **(E)** The cell viability of KYSE-30 and KYSE-180 cells measured by MTS assay. **(F)** The synthesis of DNA in were showed by BrdU incorporation assay KYSE-30 and KYSE-180 cells. **(G-H)** The colony forming assay in YES-2 and KYSE-450 cells. **(I-J)** The colony forming assay in KYSE-30 and KYSE-180 cells. GapmeR CASC8, GapmeR targeting CASC8; GapmeR control, GapmeR targeting scrambled control. Vector, cells were infected with empty lentiviruses; CASC8, cells were infected with lentiviruses expressing CASC8. Sh/CASC8, small hairpin RNA for CASC8; shRNA/NC, small hairpin RNA for negative control. ***P<0.001. All values are expressed as the mean ± SEM.

**Figure 3:**
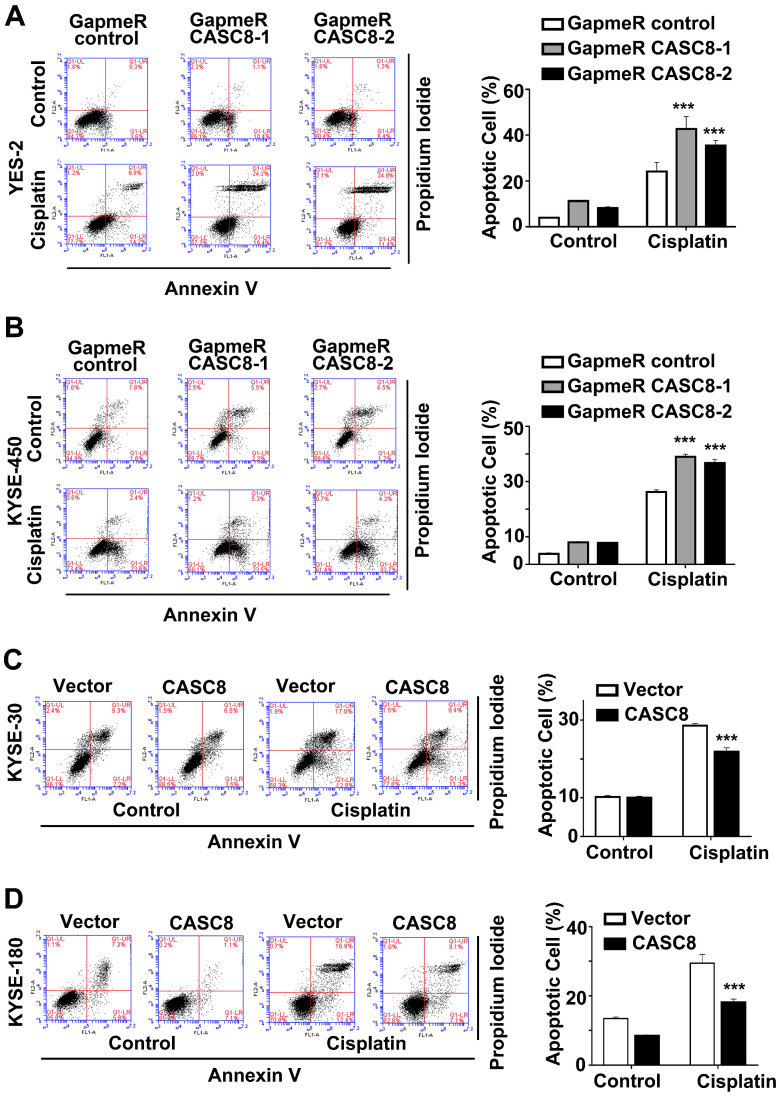
CASC8 promotes cisplatin resistance in ESCC. **(A)** The apoptotic cells in YES-2 was represented by Annexin V and PI staining using flow cytometry in left panel. Quantification of the apoptotic cells in right panel. **(B)** The apoptotic cells in YES-2 was represented by Annexin V and PI staining using flow cytometry in left panel. Quantification of the apoptotic cells in right panel. **(C)** The apoptotic cells in KYES-30 was represented by Annexin V and PI staining using flow cytometry in left panel. Quantification of the apoptotic cells in right panel. **(D)** The apoptotic cells in KYSE-180 cells was represented by Annexin V and PI staining using flow cytometry. Cells were treated with 16 μM cisplatin for 24 h. *P < 0.05, **P < 0.01, and ***P < 0.001. All values are expressed as the mean ± SEM.

As CASC8 was highly expressed in ESCC, we further explored the function of CASC8 in ESCC cells by silencing its expression with locked nucleic acid GapmeRs. GapmeRs are antisense oligonucleotides that consist of deoxynucleotide monomers, which induce sufficiently RNase H-dependent cleavage of the targeted lncRNA 23. As shown in figure 2A, transfection with CASC8-antisense GapmeRs led to a 70% reduction in transcript levels in both YES-2 and KYSE-450 cells. The cell viability and proliferation were inhibited when CASC8 was silenced in both YES-2 and KYSE-450 cells (Figure 2B and 2C). In contrast, overexpressing CASC8 in KYSE-30 and KYSE-180 cells improved cell viability and proliferation (Figure 2D-2F). We also used two independent siRNAs to knock down CASC8 expression in both YES-2 and KYSE-450 cells. These data suggested that siRNA treatment successfully inhibited the expression of nuclear-localized CASC8 (Figure S2). To further investigate the function of CASC8 during ESCC tumorigenesis, we established stable CASC8-knockdown ESCC cells using two small hairpin RNAs (shRNA), with shRNA-NC as negative control. The colony formation was decreased following CASC8 knockdown in both YES-2 and KYSE-450 cells (Figure 2G and 2H), whereas the colony formation was increased following CASC8 overexpression in both KYSE-30 and KYSE-180 cells (Figure 2I and 2J). Furthermore, CASC8 knockdown increased cisplatin-induced apoptosis in YES-2 and KYSE-450 cells (Figures 3A and 3B). On the contrary, CASC8 overexpression decreased cisplatin-induced apoptosis in both KYSE-30 and KYSE-180 cells (Figures 3C and 3D). Together, these observations indicated that CASC8 promoted cell proliferation and cisplatin resistance in ESCC.

### CASC8 promotes tumor growth *in vivo.*

**Figure 4:**
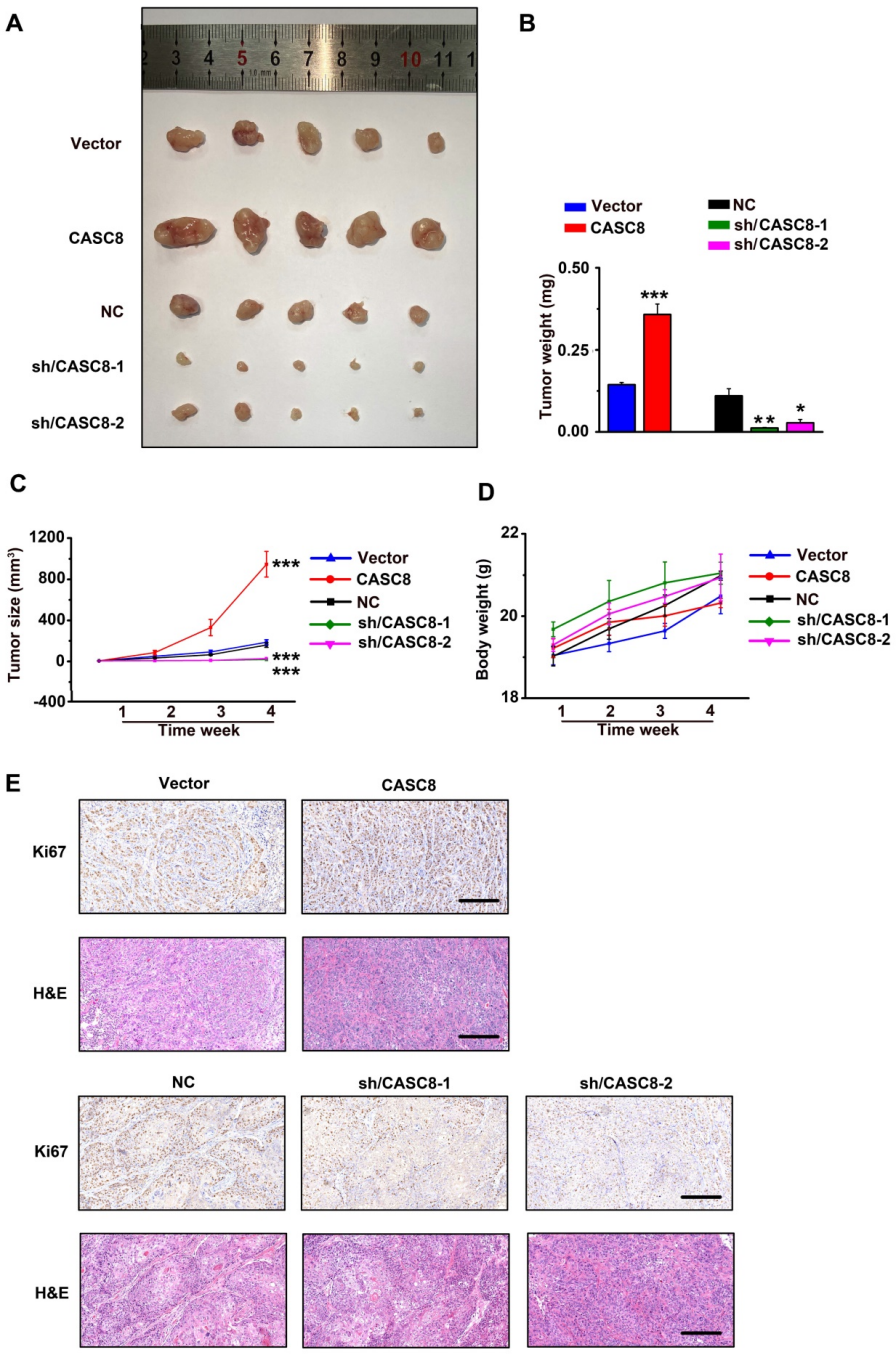
CASC8 promotes tumor growth in vivo. **(A)** The xenograft tumors from nude mice in different group. **(B)** Tumor weight of the tumors in different mice group. **(C)** Tumor growth curve and the tumor volumes of mice (n=5). **(D)** The body weight of the nude mice. **(E)** Hematoxylin and eosin staining and Ki67 staining for tumor tissues from mice. Scale bar, 50μm. *P<0.05, **P<0.01, and ***P<0.001. All values are expressed as the mean ± SEM.

Next, YES-2 cells transfected with shRNA/NC or shRNA/CASC8 and KYSE-30 cells transfected with empty vector (Vector) or vectors expressing CASC8 were subcutaneously injected into nude mice. Tumors from the shRNA-CASC8 group showed significant lower weights and smaller volumes compared with control mice (Figure 4A, 4B and 4C). On the contrary, we observed higher tumor weights and larger tumor volumes in CASC8 group compared with the vector group. Moreover, to exclude the side effect of CASC8 on physiological activities of the mice, we found no obvious body weight decrease in the mice after CASC8 knockdown or overexpression, indicating a low toxicity of CASC8 (Figure 4D). After CASC8 overexpression, the expression of Ki67, which act as a marker of proliferation, was significantly increased while the opposite results were observed after knockdown of CASC8, and the similar changes were observed in H&E staining assay (Figure 4E).

### CASC8 interacts with hnRNPL and stabilizes hnRNPL

**Figure 5:**
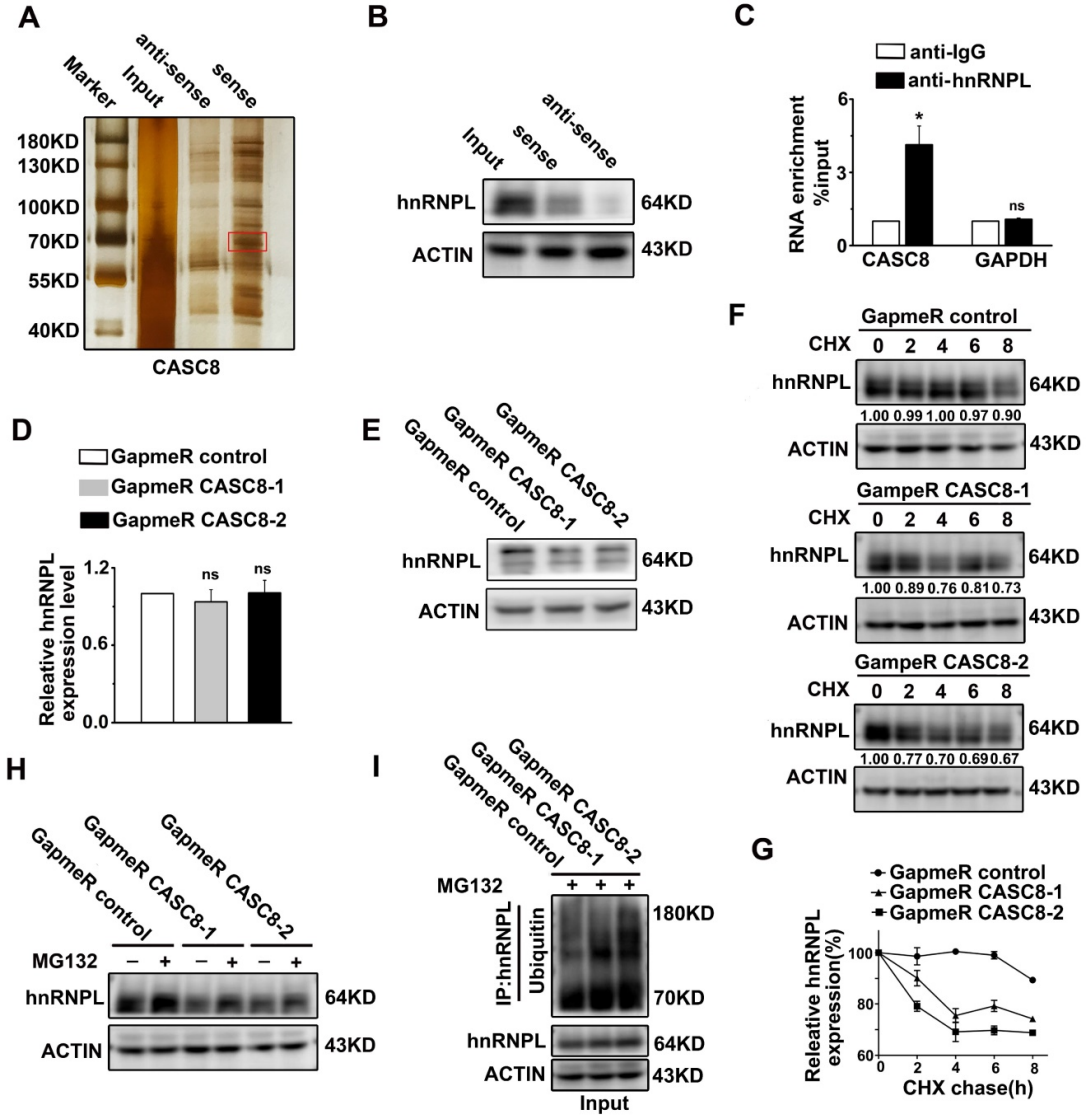
CASC8 interacts with hnRNPL. **(A)** The silver staining showed specific bands in sense CASC8 through RNA pull-down assay in KYSE-450 cells. **(B)** Western blot showed the interaction of CASC8 and hnRNPL in KYSE-450 cells. **(C)** RNA immunoprecipitation and qRT-PCR assays indicated the CASC8 enrichment with hnRNPL antibody and negative controls in KYSE-450 cells. **(D)** qRT-PCR showed the unchanged mRNA expression of hnRNPL after CASC8 knockdown in KYSE-450 cells. **(E)** Western blot analyses showed the decreased protein expression of hnRNPL after CASC8 knockdown in KYSE-450 cells. **(F)** CASC8-knockdown KYSE-450 cells were treated with 100 μg/ml CHX for 8 h and then collected at the indicated times for western blotting. **(G)** The relative levels of hnRNPL. **(H)** KYSE-450 cells transfected with GapmeR targeting CASC8 were treated with or without 10 μM MG132 for 6 h and subjected to western blot analysis. **(I)** Lysates from KYSE-450 cells that had been transfected with GapmeR targeting CASC8 and treated with MG132 were subjected to immunoprecipitation with anti-hnRNPL antibody followed by immunoblotting analysis with anti-ubiquitin or anti-hnRNPL antibody. *P<0.05. All values are expressed as the mean ± SEM.

To determine the mechanism of how CASC8 promotes ESCC, we performed RNA pull-down assay combined with silver staining to identify the CASC8-binding proteins. There was one specific band in the CASC8 pull-down samples which was excised and analyzed by mass spectrometry (Figure 5A). In the list of the mass spectrometry annotated proteins, hnRNPL was selected as a potential CASC8 interacting protein. Further validation of the binding of CASC8 to hnRNPL was confirm by western blot using the retrieved proteins from the RNA pull-down assays (Figure 5B). Moreover, RNA immunoprecipitation in KYSE-450 cells exhibited that CASC8 interacted with hnRNPL protein (Figure 5C).

To address the biological function of the interactions between CASC8 and hnRNPL, we explored the effects of CASC8 on protein and mRNA levels of hnRNPL. As shown in Figure 5D and E, CASC8 silencing decreased hnRNPL protein levels, but had not effect on hnRNPL mRNA levels, indicating that CASC8 upregulated hnRNPL protein levels at the post-transcriptional level. Considering that the ubiquitination/proteasome degradation is the common and quickest mechanism that regulated proteins stability in post-transcriptional way 24-26, we hypothesized that CASC8 upregulated hnRNPL by preventing its ubiquitination. To test whether CASC8 stabilized hnRNPL protein, we treated KYSE-450 cells with CHX, an inhibitor of protein biosynthesis, and found that CASC8 knockdown dramatically accelerated hnRNPL protein degradation (Figure 5F and 5G). To further confirm that CASC8 hinders hnRNPL protein degradation, we treated CASC8-knockdown KYSE-450 cells and control cells with MG132, a proteasome inhibitor. The result showed that cells treated with MG132 inhibited the decrease in the hnRNPL protein levels caused by knockdown of CASC8 (Figure 5H). We also performed an *in vivo* ubiquitination assay, which showed that silencing CASC8 increased hnRNPL polyubiquitination levels upon MG132 treatment (Figure 5I). Taken together, these results strongly suggested that CASC8 inhibited hnRNPL protein polyubiquitination and proteasomal degradation, thus led to increase of hnRNPL protein levels.

### CASC8 promotes ESCC progression by upregulating hnRNPL

**Figure 6:**
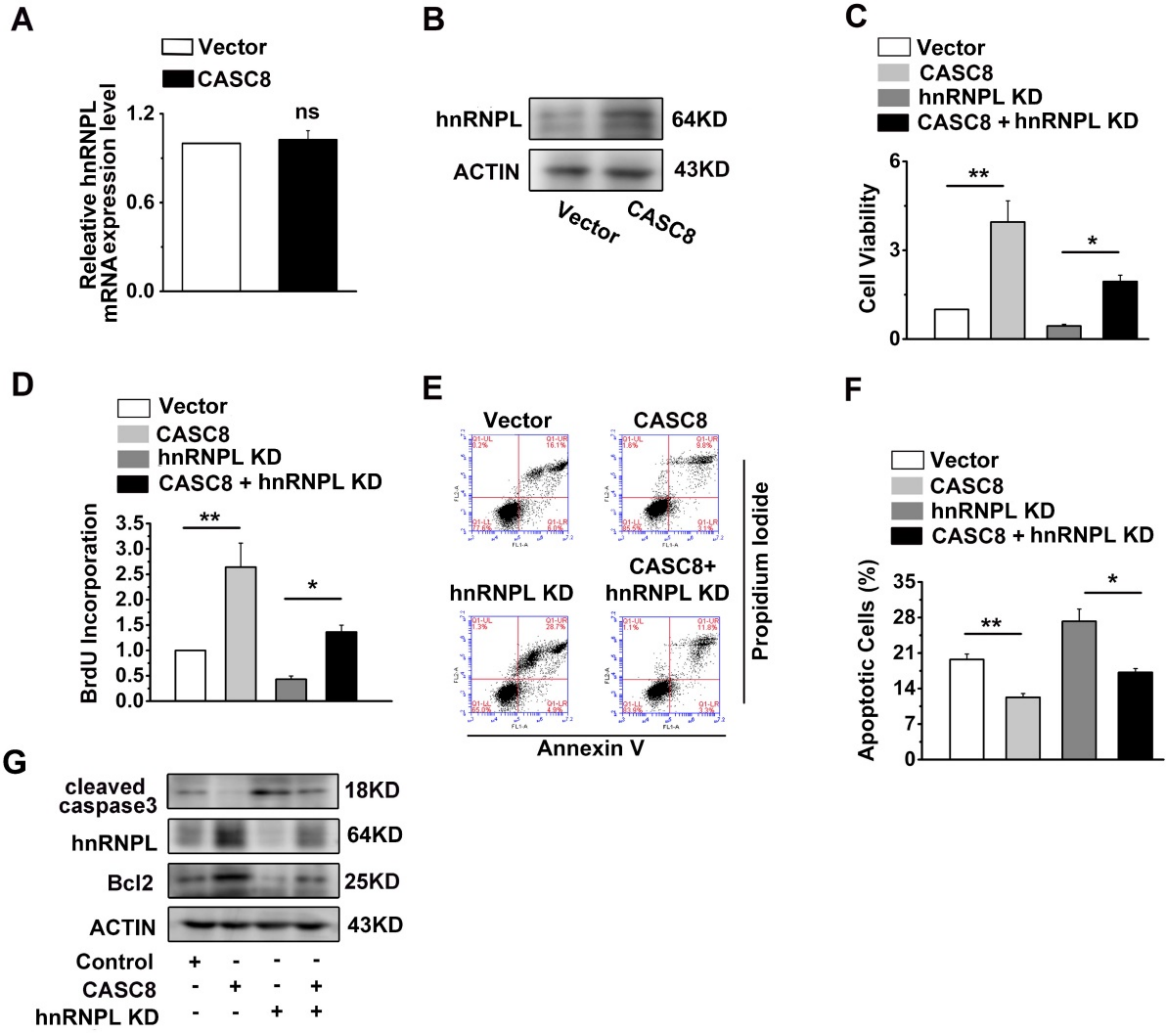
CASC8 promotes ESCC progression by upregulated hnRNPL. **(A)** Expression of hnRNPL in CASC8-overexpressed KYSE-450 cells. **(B)** The protein expression levels of hnRNPL in CASC8-overexpressed KYSE-450 cells. **(C)** The cell viability was determined by MTS assay in KYSE-450 cells. **(D)** The BrdU incorporation assay in KYSE-450 cells. **(E)** The percentage of apoptosis cells in KYSE-450 cells were determined by flow cytometry. **(F)** Quantification of the apoptotic cells. **(G)** Western blot showed the protein expression levels of Bcl2, hnRNPL, caspase3 and caspase9. *P<0.05, and **P<0.01. All values are expressed as the mean ± SEM.

We observed that CASC8 overexpression increased the protein levels of hnRNPL, while the mRNA of hnRNPL displayed little changed (Figure 6A and 6B). The improvement of CASC8 overexpression on ESCC cell proliferation was inhibited by the depletion of hnRNPL (Figure 6C and D). Furthermore, the decreased percentage of apoptotic cells caused by CASC8 overexpression was successfully rescued by si-hnRNPL (Figure 6E and F). Considering that hnRNPL is linked to the Bcl2 and caspase family, which are necessary for the initiation and execution of cell apoptosis 27-29, we also detected the expression of cleaved caspase3 and Bcl2. CASC8 overexpression was found to increase the protein levels of Bcl2 and decreased the protein levels of cleaved caspase3, while silencing hnRNPL abrogated the stimulatory effect of CASC8 overexpression (Figure 6G). Collectively, these results suggested that CASC8 promoted ESCC progression probably by enhancing hnRNPL expression.

### The ALKBH5-mediated m6A demethylation is involved in the upregulation of CASC8

**Figure 7:**
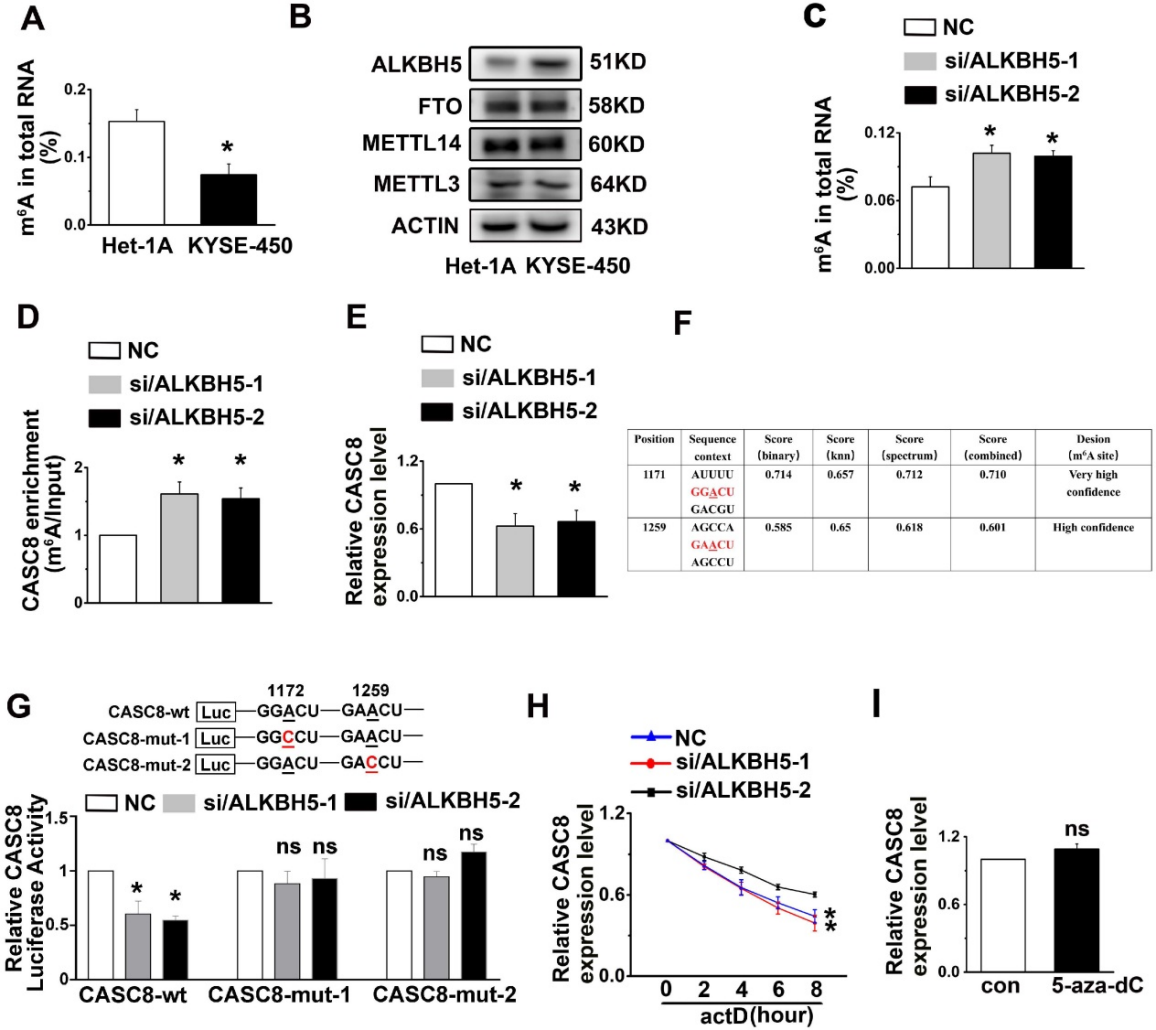
m6A modification is associated with CASC8 upregulation in ESCC cells. **(A)** Total RNA was extracted and m^6^A levels were determined as a percentage of all adenosine residues in RNA in KYSE-450 and Het-1A. **(B)** Western blot showed the protein expression levels of METTL3, METTL14, FTO and ALKBH5 in Het-1A and KYAE-450 cells. **(C)** Total RNA was extracted, and m6A content was determined as a percentage of all adenosine residues after ALKBH5 knockdown in KYSE-450. **(D)** The m^6^A level of CASC8 was measured in KYSE-450 cells with ALKBH5 knockdown in KYAE-450 cells. **(E)** CASC8 expression level was unchanged with ALKBH5 knockdown in KYSE-450 cells. **(F)** SRAMP was used to predict the m^6^A modification sequence motifs of CASC8. **(G)** The relative CASC8 luciferases activity under ALKBH5 knockdown.** (H)** KYSE-450 cells were treated with actinomycin D (5 µg/mL) and the CASC8 the expression level was measured by RT-qPCR. H. KYSE-450 cells were treated with or without 5-aza-dC (a DNA methyltransferase inhibitor), and CASC8 expression level was examined by RT-qPCR.

A number of reports in tumor epigenetic regulation have demonstrated that m6A is largely involved in modification of lncRNA 30, 31. We therefore wondered if m6A was associated with CASC8 upregulation in ESCC. First, Het-1A and KYSE-450 cells were used to detect the percentage of m6A residues among total adenosine residues in RNA. We found that the total m6A levels in RNA from Het-1A cells was higher than the levels in KYSE-450 cells (Figure 7A). We then examined the crucial m6A methyltransferase METTL3 and the m6A demethylase ALKBH5 in Het-1A and KYSE-450 cells and found that ALKBH5 was highly expressed in KYSE-450 compared to Het-1A cells, while the METTL3 expression showed no obvious changes between Het-1A and KYSE-450 cells (Figure 7B). To confirm that increased ALKBH5 expression led to decreased m6A modification of RNA in ESCC cells, we silenced the expression of ALKBH5 in KYSE-450 cells and found that ALKBH5 knockdown increased RNA demethylation in ESCC cells (Figure 7C). The m6A level in CASC8, as well as the CASC8 expression level, was also raised in ALKBH5-knockdown KYSE-450 cells compared to the control group (Figure 7D and 7E). We further identified two m^6^A modification sequence motifs in the 3'UTR of CASC8 using computation software called SRAMP (Figure 7F). To validate the m^6^A modification, we constructed a luciferase reporter inserting CASC8 wild type (CASC8-wt) or two site mutant sequence (CASC8-mut-1 and CASC8-mut-2), whose putative m6A sites were mutated. As expected, the luciferase activity of CASC8-wt cells tended to decrease when ALKBH5 was silenced, while the 1171 and 1259 site mutant groups seemed to be unchanged (Figure 7G). Considering that the m^6^A modification of the 3'UTR may affect RNA stability, an actinomycin D treatment was employed in the KYSE-450 cells to block transcription. The result displayed that the half-life of CASC8 became significantly shorter after ALKBH5 depletion (Figure 7H). Besides, we treated cells with 5-zaz-dc, a DNA methyltransferase inhibitor, to confirm whether CASC8 expression was affect by DNA methylation in ESCC. However, the CASC8 expression did no significantly changed after 5-zaz-dc treatment (Figure 7I). These findings revealed that ALKBH5-mediated m6A demethylation was associated with the upregulation of CASC8 in ESCC, probably by regulating the stability of its transcript.

## Discussion

LncRNA dysregulation have been identified as an important pathogenic factor, especially in malignancies 32-35. Several studies have reported that CASC8 exhibits high expression levels in EC which is correlated with overall survival of EC patients 36, but its function and mechanism are barely reported. In the present work, we demonstrated that lncRNA CASC8 played an important role in ESCC malignant progression. CASC8 was found to localized in the nucleus, and both gain and loss of function experiments confirmed that CASC8 could promote ESCC cell proliferation and inhibit cisplatin-induced apoptosis *in vivo* and *in vitro*.

The RNA-binding protein hnRNPL, a type of RNA binding protein, is likely to play a major role in various biological process including transcription, protein translation processing, alternative splicing and chromatin remodeling 37-40. hnRNPL has also participated in many carcinogenic processes such as proliferation, apoptosis resistance, DNA damage response and repair 37, 38, 41-43, but its mechanism is unknown. We showed here that CASC8 was an oncogene and was able to regulate the ESCC progression by directly binding to hnRNPL to protect it from ubiquitin-mediated degradation. To our knowledge, we firstly demonstrated that hnRNPL exerts an important role in the cancer development via post-transcriptional modifications.

We also tried to determine if the E3 ubiquitin ligase MDM2 interacted with hnRNPL to regulate its ubiquitination, but unfortunately the results were not significant, so we speculate that there might be other ubiquitin ligases regulating hnRNPL expression which we will explore in the future study.

Total RNA m^6^A modification plays different roles in multiple types of tumors, including glioma 44, breast cancer 45 and endometrial cancer 46, in which total RNA m^6^A hypomethylation are shown to cancer progression 47. However, two studies have shown very different results in hepatocellular carcinoma. It was reported that METTL14 was significantly downregulated in hepatocellular carcinoma, in turn reduce the total RNA m^6^A modifications and led to metastasis and invasion of hepatocellular carcinoma 48. In contrast, other researchers reported that the overexpression of METTL3 was related to the poor prognosis of hepatocellular carcinoma 49. In this study, we found that total m6A levels in RNA from KYSE-450 cells was lower than in Het-1A cells and that ALKBH5 was highly expressed in KYSE-450 compared to Het-1A cells. Furthermore, ALKBH5 knockdown increased RNA m6A modifications in ESCC cells. This is in line with the previous report that ALKBH5 is upregulated in ESCC and promotes cell proliferation.

In the recent years, the remarkable advancements have been obtained in our understanding of m^6^A modifications in influencing the RNAs 47, 50. The m6A modification participates in the progression of many diseases by targeting the normal functions of mRNA or miRNA. Nevertheless, there are a lack of studies on m^6^A modification in lncRNAs in ESCC. One recent study revealed that smoking decreased m^6^A modification of LINC00278 in ESCC 51. Herein, we demonstrated that ALKBH5 inhibited the m6A modification of CASC8, resulting regulate CASC8 RNA stability. We also confirmed that m6A methylation, but not DNA methylation in general, was involved in the upregulation of CASC8 in ESCC cells. In conclusion, our findings demonstrated the oncogenic role of lncRNA CASC8 in the progression of ESCC. We have discovered that CASC8 post-translationally regulates hnRNPL expression by protecting it from ubiquitin-mediated degradation. Furthermore, m^6^A modification may regulate CASC8 expression in ESCC cells. These results uncovered the molecular mechanism of CASC8 in the progression of ESCC, which would promote the development of novel clinical therapies.

## Supplementary Material

Supplementary figures.

## Figures and Tables

**Table 1:** Association of CASC8 Expression with clinicopathological features of 112 ESCC patients

Clinicopathological Features		Total cases (%)		CASC8 expression		P value
				High (%)	Low (%)	
Gender	Male	94(83.9)	49(52.1)		45(47.9)	0.6082
	Female	18(16.1)	11(61.1)		7(38.9)	
Age	≤ 65	66(58.9)	35(53)		31(47)	1
	> 65	46(44.1)	25(54.3)		21(45.7)	
Lymph Node Metastasis	Yes	51(45.5)	27(52.9)		24(47.1)	1
	No	61(54.5)	33(54.1)		28(45.9)	
Pathological Stage	< Ⅱ	36(32.1)	14(38.9)		22(61.1)	0.0426
	≥ Ⅱ	76(67.9)	46(60.5)		30(39.5)	
T Stage	T1+T2	27(24.1)	16(59.3)		11(40.7)	0.4963
	T3+T4	85(75.9)	44(51.8)		41(48.2)	
AJCC Stage	1	2(1.8)	2(100)		0(0)	0.6011
	2	64(57.1)	33(51.6)		31(48.4)	
	3	44(39.3)	24(54.5)		20(45.6)	
	4	2(1.8)	1(50.0)		1(50.0)	
						

## Abbreviations

ESCC: Esophageal Squamous Cell Carcinoma
CASC8: Cancer Susceptibility Candidate 8
Lnc RNA: Long noncoding RNA
HnRNPL: Heterogeneous Nuclear Ribonucleoprotein L
ALKBH5: AlkB Homolog 5
m6A: N6-methyladenosine
BrdU: Bromodeoxyuridine
FISH: Fluorescence In-Situ Hybridization
EC: Esophageal Carcinoma
