# Supplementary material for: The m6A-induced lncRNA CASC8 promotes proliferation and chemoresistance via upregulation of hnRNPL in esophageal squamous cell carcinoma
Source: Int J Biol Sci. 2022 Jul 18;18(13):4824–36. doi: 10.7150/ijbs.71234 (PMC9379415; doi:10.7150/ijbs.71234)
Supplement: Supplementary file 1 — Supplementary figures. [file ijbsv18p4824s1.pdf]

The m6A-induced lncRNA CASC8 promote proliferation and chemoresistance via upregulation of hnRNPL in esophageal squamous cell carcinoma

Supplemental figure

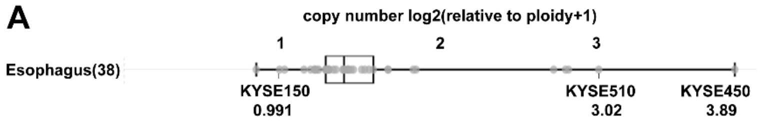

Figure S1. The CASC8 expression in esophageal carcinoma cells in CCLE database.

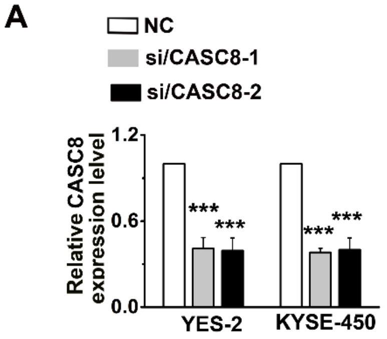

Figure S2. siRNA against CASC8 was assessed by qRT-PCR in YES-2 and KYSE-450 cells.

A

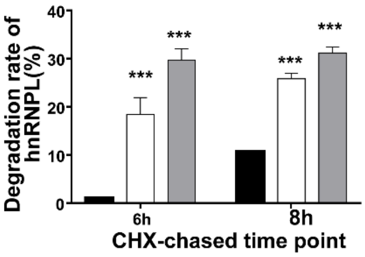

Figure S3. The degradation rates of hnRNPL in CASC8 silencing groups were significantly higher than that in control cells at 6h and 8h.
